# Reminiscences and Criticisms

**Published:** 1893-06

**Authors:** G. J. Waggoner

**Affiliations:** Larned, Kan.


### REMINISCENCES AND CRITICISMS.

G. J. WAGGONER, M. D., LARNED, KAN.

In the *Medical Advance*, September, 1880, Dr. T. P. Wilson says of Dr. Hering: "The heroic old man is dead. Here ends one of the grandest epochs of medical history. Henceforth on her fair pages shall stand four-score years marking the life and labors of Constantine Hering. What a magnificent spectacle is the production and completion of such a man! What a crown of glory it places upon the brow of humanity! What a priceless gift is such a life to the human race! What a harvest of intellectual wealth is now gathered by the death of this great man! Like some choice ceramic he passed unharmed through the fire, and became more and more beautiful as the flames of adversity burned deeply into his moral and mental nature the immortal colors of deathless life. Constantine Hering was a born leader of men because of his high intellectual endowments and his whole-souled devotion to truth. It is a supreme happiness to us who live to-day that we have seen such a spotless life completed. He lived and worshiped at the shrine of nature, and when his days were numbered he fell into the mighty arms of the All-Nature which had called him in his infancy, and in that great bosom, the love and power of which no man knows, Constantine Hering sleeps the sleep of the just."

In the somewhat remote past, when I was a private pupil of this great man, and at the same time listening to the lectures of Dr. Walter Williamson, the hearty and bluff Professor of Materia Medica in the old Filbert Street College in Philadelphia, whose chief reliance in nearly all forms of disease, at least in his own person, was Aconite, mother tincture, like the late Charles Julius Hempel, and whose other teaching in materia medica was along nearly the same line of theraputics, it may readily be seen that there was a conflict of instruction, and William A. Gardner, the genial and convivial—the idol of the school, and who betimes referred to the opinions of "our friend Dr. Jayne," with a laugh. Isaac M. Ward, the bland and complacent obstetrician, whose chief reliance was in the first and second dilutions, and almost always in alternation of two or more of them. Dr. Alvin E. Small, whose name as well as his Homœopathy was at this time a misnomer, at first of the chair of Physiology, and later of Theory and Practice, an excellent man and a good friend to all. Of Dr. Semple who presided over the laboratory little need be said, except that he was a fairly good chemist in his way. Jacob Beakley, of the chair of Surgery, I had almost forgotten. I remember him more for his rigorous methods of doing his work than for the excellence of work done. Last and not least was the renowned Fred Humphreys, the ex-presiding elder of the M. E. Church, and whom the Cleveland School charged with irregular (?) practices. At holiday time he made a visit to his home in New York, and the trustees consented to his remaining there, when my friend Dr. Small was promoted to the chair of Theory and Practice as before stated.

Here was a dilemma, indeed, for a poor ignoramus like me. While listening to this galaxy of medical lore, and at the same time receiving the instruction of my preceptor, you may readily perceive that there could be no wonder that I followed my *Alma Mater* rather than my preceptor. This was the more deplorable, inasmuch as I started all wrong, and fumed and fretted through a series of years before I discovered the fact. When I did make this discovery, I got everything in my reach that the master had ever written, and set about studying it thoroughly. Together with this I secured Hahnemann's *Materia Medica Pura* and *Chronic Diseases,* by which I began to feel my way to a better method of treating the sick. That to do so successfully I must find that remedy that had all the symptoms of the case in hand. But here again I made all my efforts, or nearly all, unavailing, by a constant repetition of the dose, until my patient was worse off than when I began. What was I to do? I felt sure that I was approaching the right track, but that there was a screw loose somewhere. All this time I had overlooked the important fact that both Hahnemann and Hering had pursued the practice of giving a single dose and waiting its full action. About this time I was taking that excellent journal, THE HOMŒOPATHIC PHYSICIAN, that has helped so many, like myself, to a better way, and to a clearer understanding of pure Homœopathy. This I read with care and avidity. Here I gathered the fruits of such minds as Lippe, Wells *et al.*, and concluded that the reason of my many failures was to be found in my oft-repeated doses. I remembered that in many cases, especially the more chronic ones, my patients would make splendid improvement for a time, and then come to a standstill, where they were wholly immovable by anything I could do. Thus I commenced in my feeble and imperfect way, in a few cases, to give a single dose and await the results, and in time was so much encouraged by the results that I adopted it in all cases, both acute and chronic, and was rewarded by better cures than I had ever dreamed of previously. Again, I began to feel my way carefully with the higher potencies, commencing with the 200th, and then the 1,000th, and finally in some cases, the CM. At this point I compared my situation with the early years of my endeavors, when, though calling myself a homœopathist, I was but a veritable mongrel fraud, and you may be sure that I felt greatly elated with the light that was beginning to dawn upon me. About this time I was called to see a diphtheritic patient, a strong, healthy man of thirty-five or forty, who had been sick three days. The family were Poles, and could scarcely speak a word of English. I found the patient with a dry, swollen tongue, protruding from the mouth, unable to speak or swallow. Had not eaten or slept for over twenty-four hours. The diphtheritic odor was appalling.

I gathered that his sore throat commenced on left side and went to the right, and that in the earlier stage he feared to go to sleep because he felt so bad on awaking. With these meagre symptoms as a guide, I ventured to prescribe Lach.$^{2c}$, one dose, of No. 20 pellets, three dry on tongue, and placebo, to be taken at intervals of two hours, then left with the understanding that I would call next morning. You may judge of my fear and trepidation when I inform you that this was my first important venture with the single remedy, and the single dose of a high potency. However, on my return the next morning, I saw a broad grin on all faces, including that of the patient. The result proved altogether favorable. My patient was well. This, with many other cases, so reassured me that I adopted these methods, and have not since departed from them. And, as the years roll by, I am more and more assured that I have found the better way. All this implies hard work and careful study; often attended by failures; but the failures become less frequent, and the better work done is an ample reward for all the pains bestowed upon it. Some few years since had occasion to treat a case of necrosis of tibia. The patient was an Englishman, of some forty years, stout and rugged, short and fat withal. There were a number of ulcers, from size of a pea to a quarter-dollar. There was intense voluptuous itching, relieved by scratching. He said he could scratch forever and enjoy it. The skin was not broken but burning followed it, and then came a crop of little vesicles. He got one dose of Sulphur$^{1m}$, and a bottle of Sac-lac. to take three or four times a day. I saw him a week later, when the improvement was very marked, and at the end of another week my patient was well, and has remained so ever since.

The past winter Le Roy B., æt. fourteen, had arthritic rheumatism, commencing in right knee, and extending to adjacent parts. Saw him on third day. Found the knee red and considerably swollen, with the whole limb very painful, especially at rest. He had not slept for two nights, and could not keep still long enough to get to sleep. The suffering was greatest when he commenced to move, but after he got warmed up a little could go pretty well. Gave one dose of Rhus-tox.$^{1m}$, and followed with Sac-lac. This was all the medicine he received, and was well and at school in less than a week.

Many similar cases could be given, but these I deem sufficient to illustrate the methods employed and the success attending them. In other words, all diseases are curable in this way provided we can make sure of the simillimum. Here we should administer the single dose, and await the results. If we have made no mistake in the selection, we will get either an aggravation or amelioration. In the former case, we must await its subsidence; and in the latter case, till the full benefit is had; and when this improvement ceases, the dose may be repeated, and not till then.

It is this that constitutes the higher medical education that is most demanded at the present time, without this no real advance can be made in the healing art. I do not decry a knowledge of the elementary branches of instruction, on the contrary believe they should be the best possible. But without a thorough and complete comprehension of the philosophy of Homœopathy, and a thorough study of the *Materia Medica*, no physician can be properly equipped for the full duty of his calling.

I freely admit my disappointment in some of the members of the I. H. A. One member gave a dose every two hours for twenty-four hours. Another, four doses at intervals of an hour in beginning each case. And others are stilt-stalkers and can hardly descend to earth long enough to know of terrestrial facts, and others ride their hobbies till they become sadly deformed. One learned gentleman, in a series of clinical cases, on the hobby plan, reported in the April number of THE HOMŒOPATHIC PHYSICIAN, was called at three A. M. to see a man who had a chill in the night, and very severe pain in his right side, the pain starting in the lower part of chest and going through to back, worse from every motion; from coughing or taking a long breath; relieved from lying on affected side * * * this pain was knife-like and was very severe; he groaned with every breath. Bryonia$^{5cm}$. "So far, so good! But wait a little." Called at ten A. M. (seven hours later), found the pain *all gone* * * * he is very stupid and drowsy, and will not keep awake long enough to get his symptoms. *Aches all over.* Bapt.$^{600m}$."

Now, why could not this gentleman have let well enough alone? It is obvious. He *had his hobby to ride.* When all this salutary change had followed the exhibition of the indicated remedy, the only thing for a rational homœopathician to do is to wait; and in all likelihood recovery would have followed the *one dose.* It strikes me that the I. H. A. has sadly fallen from its high estate. There was a time when it would have been my highest ambition to become a member of it, but that time is passed. I hope you will pardon these criticisms. I cannot refrain from making them.

